# Estimating the extent of subclinical arteriosclerosis of persons with prediabetes and diabetes mellitus among Japanese urban workers and their families: a cross-sectional study

**DOI:** 10.1186/s12872-016-0230-6

**Published:** 2016-02-24

**Authors:** Tsukasa Namekata, Kohji Shirai, Naohito Tanabe, Kunio Miyanishi, Mitsuko Nakata, Kenji Suzuki, Chikao Arai, Norio Ishizuka

**Affiliations:** Pacific Rim Disease Prevention Center, P.O.Box 25444, Seattle, WA 98165-2344 USA; Department of Health Services, School of Public Health, University of Washington, Box 357660, Seattle, WA 98195-7660 USA; Department of Vascular Function, Sakura Hospital Medical Center, Toho University, 546-1 Shizu, Sakura City, Chiba 285-8741 Japan; Department of Health and Nutrition, University of Niigata Prefecture, 471 Ebigase, Higashi-ku, Niigata City, Niigata 950-8680 Japan; Japan Health Promotion Foundation, 1-24-4 Ebisu, Shibuya-ku, Tokyo 150-0013 Japan

**Keywords:** Cardio-ankle vascular index (CAVI), Diabetes mellitus, Prediabetes, Arteriosclerosis, Epidemiology, Japanese population

## Abstract

**Background:**

Diabetes mellitus (hereafter called diabetes) is considered to accelerate arteriosclerosis leading to coronary heart disease and stroke. Thus, it is important to quantitatively estimate the extent of subclinical arteriosclerosis. A new method called cardio-ankle vascular index (CAVI) is developed to reflect arterial stiffness independently from blood pressure at the time of measurement. Then, we examined if CAVI scores could discriminate the extent of arteriosclerosis between persons with prediabetes (or borderline diabetes) and with diabetes among Japanese urban workers and their families.

**Methods:**

Subjects were 9881 men and 12033 women of company employees and their families who participated in cardiovascular disease screening in Japan. Persons having diabetes and prediabetes were defined based on the criteria set by American Diabetes Association. CAVI scores were measured by VaSera VS-1000. We applied the established age-sex specific cutoff points of CAVI scores above which were determined to be abnormally high or advanced level of arteriosclerosis. To examine the association of prediabetes and diabetes with CAVI scores, CAVI scores of screening participants were converted to a binary variable: 1 for less than cutoff points and 2 for equal or greater than cutoff points or abnormally high CAVI scores. Logistic regression method was used to examine the association of prediabetes and diabetes with CAVI scores after adjusting for major cardiovascular disease (CVD) risk factors.

**Results:**

Prevalence of abnormally high CAVI scores was significantly higher after 40 years of age among persons with diabetes than either among persons with prediabetes or among normal persons in both genders. Significantly elevated odds ratios (ORs) of abnormally high CAVI scores appeared among persons with prediabetes: 1.29 (95 % confidence interval (CI), 1.11-1.48) for men and 1.14 (CI, 1.01-1.28) for women, and among persons with diabetes: 2.41 (CI, 1.97-2.95) for men and 2.52 (CI, 1.94-3.28) for women.

**Conclusions:**

The extent of subclinical arteriosclerosis (including arterial stiffness and atherosclerosis) was moderately enhanced among persons with prediabetes and was further advanced among persons with diabetes. Thus, it is important to introduce earlier interventions for changing lifestyle and diet of persons with prediabetes in order to prevent them from developing diabetes and further advancing arteriosclerosis.

## Background

One of the most serious complications among person with diabetes is the deterioration of arterial system or the acceleration of arteriosclerosis which could not be easily measured in the past. One method to quantitatively estimate the extent of arteriosclerosis is the use of the pulse wave velocity (PWV). The idea on the association of PWV with arteriosclerosis is traced back to an experiment using artificial blood vessels conducted by Moens in 1878 [1]. Then, Bramwell and colleagues showed that PWV depends on the modulus of arterial volume elasticity by experiments in 1922-23 [1–5]. Their experimental results have been a basis for the development of the measurement device PWV-200 (Fukuda-Denshi Co., Tokyo) which measures PWV propagating through the aorta (thorax, abdomen, and part of common iliac artery) from the aortic valve to the femoral pulsation point, as described by Hasegawa in 1970 [6]. Because PWV is highly correlated with diastolic blood pressure, Hasegawa developed a nomogram showing the association between diastolic blood pressure and PWV. He proposed an adjustment to any measured PWV values at 80 mmHg. As a result, such an adjustment was built into the PWV-200 machine. This is an important step allowing clinicians and researchers to compare PWV values between individuals and between populations. Namekata et al. conducted cardiovascular disease prevention screening in Seattle and found that PWV was positively and significantly associated with aging (≥60 years of age), hypertension, diabetes, the ratio of total cholesterol to high density lipoprotein cholesterol, ex-smokers and negatively and significantly with alcohol consumption among Japanese Americans [7]. In addition, they had similar findings among Japanese urban workers [8].

To overcome some technical difficulty for measuring PWV, the cardio-ankle vascular index (CAVI) was developed as a new indicator of arteriosclerosis in 2004 [9]. CAVI scores quantitatively reflect arteriosclerosis of the aorta, femoral and tibial arteries based on Bramwell-Hill’s equation [1] and stiffness parameter [10] which is allowed to be converted from PWV propagating from the aortic valve to ankle. Some researchers proposed to use CAVI scores as an indicator of atherosclerosis. Nakamura et al. found a strong association of CAVI with the presence of severity of coronary atherosclerosis based on their ordinal logistic regression analysis [11]. Kadota et al. suggested the use of CAVI as a screening tool for atherosclerosis based on their findings from the general population study of 1,014 adults showing strong significant associations of CAVI scores with carotid intima-media thickness and with homocysteine after adjustment for age and sex [12]. Thus, it is considered that CAVI scores reflect arterial stiffness, atherosclerosis and arteriosclerosis of which conditions are overlapping and inseparable. We use CAVI scores to represent the extent of subclinical arteriosclerosis in this paper but it is inclusive of arterial stiffness and atherosclerosis.

To practically use CAVI as a diagnostic tool for determining the extent of arteriosclerosis, our previous study established the baseline CAVI scores by age and gender among cardiovascular disease (CVD) risk-free persons [13]. In our present study we measured CAVI scores and identified persons with abnormally high CAVI scores in Japanese urban workers and their families based on the criteria developed by our studies [13, 14], and then examined the extent of subclinical arteriosclerosis among persons with prediabetes and among persons with diabetes mellitus after making adjustment for major cardiovascular disease (CVD) risk factors.

## Methods

### Subjects

Subjects for the study were recruited from January 2006 to May 2009 through the screening program at Japan Health Promotion Foundation which has been conducting cardiovascular disease and cancer screening throughout major cities in Japan. Subjects were company employees and their family members: 9,881 men and 12,033 women between 17 and 87 years of age. Following Japan’s Personal Information Protection Law, the use of anonymized screening data for research purpose was approved by the board of Japan Health Promotion Foundation. The study was approved by the board of Pacific Rim Disease Prevention Center.

### Definition of prediabetes and diabetes mellitus

Following the recommendation from American Diabetes Association [15], persons having diabetes were defined as those having medical history of diabetes and/or taking medication of diabetes and/or whose fasting plasma glucose were equal to or higher than 126 mg/dl and/or whose hemoglobinA1c (HbA1c) were equal to or higher than 6.5 % in NGSP value (48 mmol/mol in IFCC). Persons with the state of prediabetes were defined as those whose fasting plasma glucose was from 100 mg/dl to 125 mg/dl and/or whose HbA1c was from 5.7 % to 6.4 % in NGSP value (39 mmol/mol to 47 mmol/mol in IFCC). Persons defined as “normal” were those without diabetes and prediabetes.

### Clinical measurements

Blood was drawn from subjects after a 12 h fast. The following measurements were made: total cholesterol (TC) and triglycerides (TG) by enzymatic assay; high density lipoprotein cholesterol (HDL-C) by modified enzymatic method; glucose by hexokinase glucose-6-phosphate dehydrogenate assay; and glyco-hemoglobin A1c (HbA1c) by latex agglutination.

Following the guideline released by American Heart Association in 2007 [16], persons having hypertension were defined as those having medical history of hypertension and/or taking hypertension drugs and/or whose systolic blood pressure (SBP) was equal to or higher than 140 mmHg and/or whose diastolic blood pressure (DBP) was equal to or higher than 90 mmHg.

### Cardio-ankle vascular index

CAVI, a stiffness and arteriosclerosis indicator of thorax, abdomen, common iliac, femoral and tibial arteries, was measured by VaSera VS-1000 manufactured by Fukuda-Denshi Company, LTD (Tokyo, Japan).

As illustrated by Shirai et al. [9], the scale conversion from PWV to CAVI is performed by the following formula:

$$ \mathrm{CAVI}=\mathrm{a}\left\{\left(2\uprho /\Delta \mathrm{P}\right)\ \mathrm{x}\  \ln \left(\mathrm{P}\mathrm{s}/\mathrm{P}\mathrm{d}\right){\mathrm{PWV}}^2\right\} + \mathrm{b} $$

where Ps and Pd are systolic and diastolic blood pressure values, respectively, PWV is the pulse wave velocity between heart and ankle, ∆P is Ps-Pd, ρ is blood density, and a and b are constants. This equation was derived from Bramwell-Hill’s equation [1] and stiffness parameter [10]. Scale conversion constants are determined so as to match CAVI with PWV by Hasegawa’s method [6]. These measurements and calculations are automatically made in VaSera VS-1000.

In the previous study we established the age-sex specific cutoff points of CAVI scores above which were determined to be abnormally high or advanced level of arteriosclerosis [14]. The cutoff points are (mean of CAVI + one standard deviation ) among CVD risk-free subjects: 7.39 for 20-29 years of age, 7.80 for 30-39 years of age, 8.29 for 40-49 years of age, 8.83 for 50-59 years of age, 9.54 for 60-69 years of age, and 10.35 for 70 years of age and over among men; and 7.23 for 20-29 years of age, 7.42 for 30-39 years of age, 7.95 for 40-49 years of age, 8.52 for 50-59 years of age, 8.98 for 60-69 years of age, and 9.46 for 70 years of age and over among women. To apply the logistic regression method for examining the association of prediabetes and diabetes with CAVI scores, CAVI scores of screening participants were converted to a binary variable: 1 for less than cutoff points and 2 for equal or greater than cutoff points or abnormally high CAVI scores.

### Questionnaire

A short self-administered questionnaire was filled out by each subject during the screening. It contains questions on medical history and lifestyle factors such as smoking habit and alcohol consumption.

### Statistical methods

All statistical analyses were performed gender-specifically. To examine characteristics of study participants by diabetes status, Student’s t-tests and chi-square tests were conducted for detecting significant differences in means and in prevalence, respectively, between persons with normal status and persons with prediabetes and diabetes. Cochran-Armitage test for linear trend was applied to evaluate the dose-dependent association between the degree of glycemic status and the prevalence of abnormally high CAVI scores. Crude, age-adjusted and multivariable-adjusted odds ratios (OR) and the 95 % confidence intervals (CI) of abnormally high CAVI scores according to diabetic status were calculated in logistic regression models, with normal persons who were treated as the reference category. In the age-adjusted model, age was entered as a variable of 10-year interval categories (50-59, 60-69, 70+ vs. <50). The multivariable model was further adjusted for major CVD risk factors including hypertension (yes vs. no), HDL-C (≥40 mg/dl vs. <40 mg/dl for males, ≥50 mg/dl vs. <50 mg/dl for females), triglycerides (150-199 mg/dl, ≥200 mg/dl vs. <150 mg/dl), BMI (20-22.9, 23-24.9, 25-27.9, 28-29.9, 30+ vs. <20), drinking habit (≤4 times/week, ≥5 times/week vs. non-drinkers), and smoking habit (ex-smokers, current smokers vs. non-smokers).

## Results

Characteristics of study participants by age and gender were described in our previous paper [14]. We briefly summarize those here. It is observed that the averages for both systolic and diastolic blood pressure and CAVI scores linearly increased as ages advanced in both genders with an exception of men’s diastolic blood pressure after 60 years of age of which averages slightly decreased. Almost all averages of clinical indicators increased until 60 years of age except HDL-C of which averages were at the same level in both genders. Striking differences in averages of clinical indicators between genders are observed and are unfavorable for men in terms of cardiovascular disease risk. BMI averages ranged from 22.3 to 24.2 among men and from 20.4 to 22.9 among women. Greater prevalence of drinkers and smokers was observed in men than in women. Prevalence of drinkers and smokers was greater in younger women than in older women.

Table 1 shows characteristics of study participants by diabetes status. Averages of all variables in both genders were lowest in normal, highest in diabetes, and middle in prediabetes, except averages of HDL-C which were in the reverse order. Prevalence of abnormally high CAVI scores was lowest in normal, highest in diabetes, and middle in prediabetes.

**Table 1:** Characteristics of study participants by diabetes status

		Normal	Prediabetes	Diabetes
Sample size	Men	7,202	2,001	678
	Women	8,947	2,756	330
		mean ± SD (t-value)		
Age	Men	43 ± 13	51 ± 10 (t = 28.11***)	56 ± 10 (t = 26.72***)
	Women	44 ± 12	51 ± 9 (t = 27.54***)	55 ± 9 (t = 16.60***)
Systolic blood pressure	Men	125 ± 14	131 ± 15 (t = 15.12***)	136 ± 18 (t = 18.53***)
	Women	118 ± 15	125 ± 15 (t = 21.58***)	132 ± 18 (t = 16.43***)
Diastolic blood pressure	Men	76 ± 11	81 ± 11 (t = 17.26***)	82 ± 11 (t = 14.03***)
	Women	70 ± 10	73 ± 11 (t = 14.32***)	77 ± 12 (t = 11.19***)
Total cholesterol (mg/dl)	Men	205 ± 35	215 ± 35 (t = 8.07***)	214 ± 37 (t = 4.54***)
	Women	209 ± 37	225 ± 38 (t = 18.34***)	230 ± 40 (t = 9.21***)
HDL-C (mg/dl)	Men	61 ± 17	60 ± 17 (t = 3.90***)	57 ± 18 (t = 5.81***)
	Women	77 ± 18	75 ± 19 (t = 3.63***)	70 ± 18 (t = 6.66***)
Triglycerides (mg/dl)	Men	129 ± 112	156 ± 139 (t = 8.96***)	170 ± 133 (t = 8.82***)
	Women	78 ± 52	95 ± 56 (t = 14.18***)	121 ± 80 (t = 14.30***)
CAVI	Men	7.43 ± 0.99	7.97 ± 0.96 (t = 21.28***)	8.49 ± 1.11 (t = 26.08***)
	Women	7.24 ± 0.92	7.62 ± 0.89 (t = 18.93***)	8.09 ± 1.02 (t = 16.38***)
Body Mass Index (kg/m^2^)	Men	23.5 ± 3.2	24.6 ± 3.3 (t = 13.56***)	25.1 ± 3.9 (t = 12.46***)
	Women	21.3 ± 3.0	22.5 ± 3.6 (t = 17.79***)	24.3 ± 4.2 (t = 17.53***)
		Prevalence (%) (chi-square statistics)		
Abnormally high CAVI score	Men	12.8	18.5 (χ^2^ = 43.41***)	30.4 (χ^2^ = 157.25***)
	Women	15.2	18.9 (χ^2^ = 21.01***)	34.2 (χ^2^ = 85.95***)
Drinkers	Men	74.3	77.9 (χ^2^ = 10.62**)	72.7 (χ^2^ = 0.81)
	Women	43.1	36.9 (χ^2^ = 34.05***)	23.0 (χ^2^ = 52.65***)
Ex-smokers	Men	23.3	29.5 (χ^2^ = 33.23***)	31.7 (χ^2^ = 24.29***)
	Women	9.4	7.6 (χ^2^ = 35.23***)	9.1 (χ^2^ = 10.24**)
Smokers	Men	46.0	43.1 (χ^2^ = 33.23***)	41.4 (χ^2^ = 24.29***)
	Women	12.8	9.4 (χ^2^ = 35.23***)	7.0 (χ^2^ = 10.24**)

Note: Student’s t-tests and chi-square tests were conducted by comparing means and prevalence respectively between persons with normal status and persons with prediabetes or persons with diabetes

*p*-value: *< 0.05, **< 0.01, ***< 0.001

Prevalence of abnormally high CAVI scores is shown according to the status of prediabetes and diabetes by age and gender in Table 2. It is observed that such prevalence was higher after 40 years of age among persons with diabetes than either among persons with prediabetes or among normal persons in both genders.

**Table 2:** Prevalence (%) of abnormally high CAVI scores by status of prediabetes and diabetes mellitus

	Age	≤29	30-39	40-49	50-59	60-69	70+
Men							
Normal	prevalence	6.0	10.9	13.2	21.5	12.8	11.8
	persons at risk	1042	2338	1731	1235	704	152
Prediabetes							
	prevalence persons at risk	9.1 22	16.3 270	15.5 555	23.3 738	16.9 350	12.1 66
Diabetes							
	prevalence persons at risk	― 2	11.8 51	19.1 110	43.7 263	26.9 193	20.3 59
χ^2^value for linear trend (*p*-value)		__	4.25 (0.039)	4.31 (0.038)	39.9 (0.000)	21.16 (0.000)	2.14 (0.144)
Women							
Normal	prevalence	5.9	14.8	11.6	19.8	23.2	33.9
	persons at risk	892	2657	2340	2235	711	112
Prediabetes							
	prevalence persons at risk	― 11	16.9 413	12.4 728	21.9 1184	23.3 361	30.5 59
Diabetes							
	prevalence persons at risk	― 2	― 19	20.3 59	37.7 159	42.1 76	―15
χ^2^value for linear trend (*p*-value)		__	__	2.12 (0.145)	17.53 (0.000)	6.24 (0.012)	__

Note: ― indicates that the sample size was too small to obtain meaningful prevalence

Table 3 shows odds ratios (ORs) of abnormally high CAVI scores in association with prediabetes and diabetes as compared with the reference of normal CAVI scores: (1) without an adjustment for confounding factors (crude ORs); (2) age breakdowns were added to logistic regression analysis; and (3) other CVD risk factors were further added to logistic regression analysis. As more confounding factors were adjusted, odds ratios decreased in both prediabetes and diabetes mellitus except ORs of prediabetes in women indicating that ORs after adjusting for ages and for CVD risk factors were almost identical and lower than crude OR. After adjusting for major CVD risk factors, significantly elevated ORs appeared among persons with prediabetes: 1.29 (95 % confidence interval (CI), 1.11-1.48) for men and 1.14 (CI, 1.01-1.28) for women, and among persons with diabetes: 2.41 (CI, 1.97-2.95) for men and 2.52 (CI, 1.94-3.28) for women.

**Table 3:** Estimated risk of having abnormally high CAVI scores in association with prediabetes and diabetes mellitus among Japanese urban workers and their families

	Prediabetes				Diabetes mellitus			
	OR		95 % CI		OR		95 % CI	
			lower	upper			lower	upper
Men								
(1) Crude	1.56	***	1.36	1.78	2.98	***	2.50	3.56
(2) Adjusted for age	1.32	***	1.15	1.51	2.38	***	1.97	2.88
(3) Adjusted for CVD risk factors	1.29	**	1.11	1.48	2.41	***	1.97	2.95
Women								
(1) Crude	1.30	***	1.16	1.45	2.90	***	2.29	3.66
(2) Adjusted for age	1.12		0.99	1.25	2.19	***	1.72	2.78
(3) Adjusted for CVD risk factors	1.14	*	1.01	1.28	2.52	***	1.94	3.28

Note: *OR* Odds ratio, *CI* Confidence interval; *p < 0.05, **p < 0.01, ***p < 0.001; (1) Prediabetes or diabetes was included alone as a covariate in logistic regression analysis. (2) Age breakdowns (<50, 50-59, 60-69, ≥70 years of age) were added to logistic regression analysis. (3) Other CVD risk factors (hypertension, HDL-C, TG, BMI, drinking, and smoking) were further added to logistic regression analysis

## Discussion

In the present cross-sectional study of Japanese urban workers and their families, prevalence of abnormally high CAVI scores were dose-dependently elevated along with the advancing degree of diabetic status both in men and in women. Compared with normal persons, significantly elevated odds ratios of abnormally high CAVI scores were observed not only in persons with diabetes but also in those with prediabetes.

With regard to the validity to use CAVI scores as an indicator of arteriosclerosis, Otsuka examined 72 deceased patients’ antemortem PWV (which is a basis for deriving CAVI scores) and pathological changes measured by the diffuse fibrotic thickening, formation of atheroma and calcification in the wall of their aorta. He reported multiple regression coefficient R = 0.810 between PWV and scores of those pathological changes [17]. In addition, other researchers reported that CAVI scores were significantly associated with coronary atherosclerosis [11], with carotid intima-media thickness and with homocysteine [12]. Thus, the use of CAVI scores derived from PWV values is valid to estimate the extent of arteriosclerosis.

VaSera VS-1000, which was used in our study, was designed to measure CAVI scores independent of blood pressure at the time of CAVI score measurement and CAVI scores represent the extent of arteriosclerosis between the aortic valve and the ankle. We have shown biological aging of the major artery by measuring CAVI scores in the CVD risk-free group and disease-related pathological aging of the major artery in the CVD high-risk group [13]. CAVI scores allow us to evaluate the extent of arteriosclerosis in the major arteries between the aortic valve and the ankle, to screen persons with subclinical stage of CVD, and provide an opportunity to modify diet and lifestyle to improve CAVI scores as reported by Satoh et al [18]. Thus, the use of CAVI scores potentially leads to savings on high treatment costs and to prolonging many productive lives.

Namekata et al. showed significant odds ratios of the abnormally high aortic pulse wave velocity (PWV, an indicator of arteriosclerosis reflecting stiffness of artery and atherosclerosis) in an association with diabetes among Japanese Americans and among native Japanese (3.66 and 2.43, respectively) [8]. Namekata et al. observed that odds ratios of abnormally high CAVI scores in an association with diabetes after making adjustment for ages were 10.02 for men and 8.42 for women, compared with those who did not have diabetes [14]. In our recent study significant ORs of prediabetes and diabetes were observed in association with most CVD risk factors including age, hypertension, triglycerides ≥ 200 mg/dl and BMI ≥ 25 in both genders [19]. Our results imply much faster advancement of arteriosclerosis among persons both with prediabetes and with diabetes than in the normal persons.

In our present study it is shown that prevalence of abnormally high CAVI scores has an increasing trend with an age increase in all three groups of non-diabetes normal persons, prediabetes and diabetes (Table 2). Our results show that comparing with persons with normal CAVI scores, estimated risks of abnormally high CAVI scores in association with prediabetes and with diabetes were 1.29 times higher and 2.41 times higher, respectively, in men and were 1.14 times higher and 2.52 times higher, respectively, among women (Table 3). Our study results have confirmed the association between prediabetes and arteriosclerosis and the much stronger association between diabetes and arteriosclerosis estimated by CAVI scores.

A limitation of this study is that it is an observational and cross-sectional study. Thus, we cannot tell how many persons with prediabetes will develop diabetes or will return to normal in their blood glucose levels or/and HbA1c levels. The strengths of this study are having the large sample size and being able to adjust for many CVD risk factors with enough statistical power to examine the association of CAVI scores with prediabetes and diabetes mellitus.

## Conclusion

In conclusion, both prediabetes and diabetes mellitus were significantly associated with CAVI scores. It is implied that the extent of arteriosclerosis (including arterial stiffness and atherosclerosis) was moderately enhanced among persons with prediabetes and was further advanced among persons with diabetes. Thus, it is important to introduce earlier interventions for changing lifestyle and diet of persons with prediabetes in order to prevent them from developing diabetes and further advancing arteriosclerosis.

## Abbreviations

BMI: Body mass index
CAVI: Cardio-ankle vascular index
CHD: Coronary heart disease
CI: Confidence interval
CVD: Cardiovascular disease
DBP: Diastolic blood pressure
Diabetes: Diabetes mellitus
HbA1c: Glyco-hemoglobin A1c
HDL-C: High density lipoprotein cholesterol
ORs: Odds ratios
PWV: Pulse wave velocity
SBP: Systolic blood pressure
SD: Standard deviation
TC: Total cholesterol
TG: Triglycerides
